# The Activation of ERK1/2 and JNK MAPK Signaling by Insulin/IGF-1 Is Responsible for the Development of Colon Cancer with Type 2 Diabetes Mellitus

**DOI:** 10.1371/journal.pone.0149822

**Published:** 2016-02-22

**Authors:** Jia-An Teng, San-Gang Wu, Jia-Xin Chen, Qiang Li, Fang Peng, Zhou Zhu, Jian Qin, Zhen-Yu He

**Affiliations:** 1 Department of Cadre Medicine, Division of Endocrinology and Metabolism, People’s Hospital of Guangxi Zhuang Autonomous Region, Nanning, China; 2 Department of Radiation Oncology, Xiamen Cancer Center, the First affiliated Hospital of Xiamen University, Xiamen, China; 3 Department of Radiation Oncology of Clinical Cancer Center, the People’s Hospital of Guangxi Zhuang Autonomous Region, Nanning, China; 4 Organ Transplantation Center, Department of Surgery, the Second Xiangya Hospital, Central South University, Changsha, China; 5 Department of Radiation Oncology, the First Affiliated Hospital of Sun Yat-sen University, Guangzhou, China; 6 Department of Gastrointestinal and Peripheral Vascular Surgery, the People’s Hospital of Guangxi Zhuang Autonomous Region, Nanning, China; 7 Sun Yat-sen University Cancer Center, State Key laboratory of Oncology in South China, Department of Radiation Oncology, Collaborative Innovation Center of Cancer Medicine, Guangzhou, China; Wayne State University School of Medicine, UNITED STATES

## Abstract

Previous studies showed that type 2 diabetes mellitus (T2DM) is linked to increased risk of developing colon cancer. Insulin and insulin-like growth factor 1 (IGF-1) are increased in patients with T2DM. The increased insulin and IGF-1 may be responsible for the developing of colon cancer. In this study, we investigated the effects and mechanisms of insulin and IGF-1 in colon cancer development *in vitro* and *in vivo*. Insulin and IGF-1 alone or together elevated proliferation and reduced apoptosis in colon cancer MC38 cells. Meanwhile, insulin and IGF-1 promoted the phosphorylation of extracellular-signal regulated kinase 1/2 (ERK1/2) and c-Jun N-terminal kinase (JNK). Treatment with ERK1/2 or JNK inhibitor in the presence of insulin and IGF-1 significantly decreased B-cell lymphoma 2 (Bcl-2) and increased Bcl-2-associated X protein (Bax) expression and finally increased apoptosis and inhibited the proliferation. Accelerative colon tumor growth was found in a mouse model of T2DM with *db/db* mice which got high level of endogenous insulin and IGF-1. Furthermore, the inhibition of ERK1/2 or JNK suppressed the development of colon tumor *in vivo*. These results suggest that the activation of ERK1/2 and JNK signaling by insulin and IGF-1, at least in part, is responsible for the development of colon cancer with T2DM.

## Introduction

In the past decades, due to the rapid economic growth and changes in lifestyle in China, the incidence and cases of diabetes have been rising rapidly. In China, the prevalence of diabetes was 9.7%, accounting for 92.4 million individuals living with diabetes, mainly type 2 diabetes mellitus (T2DM) [1]. T2DM is associated with an increased incidence of cancer mortality [2, 3]. In particular, the risk of breast [4], pancreas [5], liver [6] and colon [7, 8] cancer is increased in patients with T2DM. T2DM increases the lifetime risk of colon cancer by up to three times than the general population, which makes colon cancer one of the most common cancers in patients with T2DM [9].

The mechanisms underlying the connection of T2DM with colon cancer are complicated and not yet fully elucidated. T2DM is associated with hyperinsulinemia and elevated insulin-like growth factor 1 (IGF-1). Insulin, an important growth factor acting as a cell mitogen, when at high concentrations increase the risk of colon cancer by promoting growth of tumors [10]. And the insulin treatment may further elevate risk for colon cancer in patients with T2DM [11]. Insulin stimulates cell proliferation through direct binding to insulin receptors or IGF-1 receptors, or by inhibition of IGF-binding proteins, which increases IGF-1 availability to the IGF receptor [12]. The insulin or IGF-1 signaling is proved as a potent growth regulator linked with carcinogenesis in different cell lines [13]. Previous studies showed an association between elevated IGF-1 and the risks of colon cancer [14, 15]. The correlation between insulin or IGF-1 and the risks of colon cancer in patients with T2DM is accepted widely [16, 17]. Mitogen-activated protein kinase (MAPK) signal contains 3 major pathways: extracellular regulated protein kinases 1/2 (ERK1/2), c-Jun N-terminal kinase (JNK) and P38 signals. Previous studies showed that MAPK signal, which acts as downstream of insulin and IGF-1 proliferative signaling, plays a role in the proliferation of various cancers, including breast, colon and prostate cancer [18–20]. However, due to the lack of ideal colon cancer model with T2DM, it is still not clear how does this signal regulate the proliferation of colon cancer in a T2DM environment.

This study was aimed to determine the mechanism of insulin/IGF-1 in colon cancer growth within a T2DM environment. To this end, a mouse derived colon cancer cell line—MC38 cells with insulin/IGF-1 treatment and a mouse spontaneous T2DM model with tumor allografts were used to mimic the T2DM environment *in vitro* and *in vivo*. Insulin/IGF-1 elevated proliferation and reduced apoptosis in MC38 cells. Meanwhile, the effects of insulin/IGF-1 in MC38 cells were ERK1/2 and JNK dependent. Accelerative colon tumor growth was found in a mouse model of T2DM which got high level of insulin and IGF-1. Thus, we suggest that the activation of ERK1/2 and JNK signaling by insulin and IGF-1, at least in part, is responsible for regulating the development and formation of colon cancer with T2DM.

## Materials and Methods

### Cell culture

Colon cancer MC38 cell line was originally derived from C57BL/6 mice treated with the carcinogen 1,2-dimethylhydrazine [21], it was purchased from American Type Culture Collection (ATCC, Manassas, VA) and grown in DMEM medium supplemented with 10% fetal bovine serum, 1% glutamine and 1% of penicillin/streptomycin. The cells were maintained at 37°C and 5% CO_2_ in a humid environment. Medium was replaced every 48 hours and cells were trypsinized when reached 80% confluence. The cells were serum-starved overnight and then treated with 5 ng/ml or 50 ng/ml concentrations of insulin (Sigma, St. Louis, MO) and IGF-1 (Cell Signaling Technology, Beverly, MA) dissolved in phosphate buffered saline (PBS). In ERK1/2 or JNK inhibiting assays, 40 μM ERK1/2 inhibitor PD98059 (Sigma, St. Louis, MO) or 20 μM JNK inhibitor SP600125 (Sigma, St. Louis, MO) dissolved in dimethyl sulfoxide (DMSO) were added into cultured cells.

### Cell proliferation assay

Cell proliferation was determined using a cell counting kit-8 (CCK-8) (Dojindo, Kumamoto, Japan) according to the manufacturer’s protocol. Briefly, MC38 cells were seeded on 96-well plates at 8000 cells per well. After overnight incubation, the cells were treated with 50 ng/ml insulin and 50 ng/ml IGF-1 with or without 40 μM ERK1/2 inhibitor PD98059 or 20 μM JNK inhibitor SP600125 for 24, 48 or 72 hours. Then 10 μL 2-(2-methoxy-4-nitrophenyl)-3-(4-nitrophenyl)-5-(2,4-disulfophenyl)-2H-tetrazolium monosodium salt (WST-8) was added to each well and incubated at 37°C for another 2 hours. Then the optical absorbance at wave length 450 nm was measured using a microplate reader (Thermo Scientific, Waltham, MA).

### Cell cycle analysis

For *in vitro* cell cycle analysis, MC38 cells with different treatments for 72 hours were harvested and fixed with 70% ice-cold ethanol for 4 hours. Then the fixed cells were incubated with 400 μl 0.5 mg/ml propidium iodide (PI) for 30 min at 4°C. The stained cells were analyzed with a FACS Calibur (BD, Franklin Lakes, NJ). Quantification of fluorescence was done by flow cytometry to determine the cell cycle kinetics of MC38 cells in G0/G1-phase, S-phase and G2/M-phase.

### Cell apoptosis analysis

Apoptosis rate were evaluated on 7^th^ day of culture by Annexin V-FITC Apoptosis Detection Kit I (BD, San Diego, CA) according to the manufacturer’s protocol. Briefly, 1×10^6^ trypsinized cells were washed by PBS and re-suspended in Annexin V binding buffer. The cells were stained with 5 μl of FITC Annexin V and 5 μl PI for 15 min at room temperature in the dark. The stained cells were analyzed by FACS Calibur (BD, Franklin Lakes, NJ) within 1 hour.

### Western Blot analysis

MC38 cells or tumor tissues lysates were prepared using western lysis buffer containing protease and phosphatase inhibitors on ice. Cell extracts were centrifuged at 12,000 rpm for 15 min at 4°C and the supernatant was used for western blotting. Protein concentration was measured by Bio-Rad assay using the manufacturer’s protocol (Bio-Rad Laboratories, Hercules, CA). Twenty μg of supernatant proteins were separated in 10% SDS-PAGE and transferred onto polyvinylidene fluoride membranes (Millipore, Billerica, MA) for 1 hour. The membranes were blocked by 5% nonfat dry milk in Tris buffered saline containing 0.05% Tween-20 for 2 hours, followed by incubation with primary antibodies overnight at 4˚C. The primary antibodies of GAPDH (Cat#4695s), ERK1/2 (Cat#5174), p-ERK1/2 Thr202/Tyr204 (Cat#4370), JNK (Cat#9252), p-JNK Thr183/Tyr185 (Cat#9251), P38 (Cat#8690), p-P38 Thr180/Tyr182 (Cat#4511) and Caspase3 (Cat#9664) were from Cell Signaling Technology (Beverly, MA), Bcl-2 (Cat# sc-509), Bax (Cat# sc-20067) and Cyclin D1 (Cat# sc-753) were from Santa Cruz Biotechnology (Santa Cruz, CA). The membrane was then incubated with horseradish peroxidase-conjugated rabbit or mouse secondary antibodies for 1 hour at room temperature. Immunoreactive bands were detected using Super Signal West Pico chemiluminescent substrate (Thermo Scientific, Rockford, IL).

### Tumor growth studies in mouse model of type 2 diabetes

This project was approved by the Animal Care and Use Committee of the People's Hospital of Guangxi Zhuang Autonomous Region, Nanning, China (#2011–005). Male B6.BKS-Lepr^db^ mice (*db/db*) and their heterozygous littermates (*db/+*) were purchased from The Jackson Laboratory (Bar Harbor, ME). All procedures followed the National Institutes of Health guidelines for the care and use of laboratory animals. Mice were housed in a specific pathogen free facility under standard 12 hours light/dark cycle and fed standard rodent chow and water *ad libitum*. Eight-week-old *db/db* mice were used as type 2 diabetes model while the *db/+* mice as healthy controls. MC38 cells were expanded in DMEM medium supplemented with 10% fetal bovine serum and 1% of penicillin/streptomycin *in vitro*. 2 × 10^6^ MC38 cells suspended in 0.1 ml of PBS were subcutaneously injected into the right flank of each mouse to initiate tumor growth. For the *in vivo* ERK1/2 and JNK blocking studies, 10 mg/kg PD98059 or 30 mg/kg SP600125 was administered intraperitoneally (i.p.) every 3 days when tumor volume reached 100mm^3^ (7–8 days after the tumor initiation) and lasted for another 2 weeks. 1% DMSO was used as control treatment. The sizes of tumors were measured every 3 days with calipers when the tumors started growing. Any one dimension of the tumor exceeded 20 mm or the tumor burden was greater than 10% body weight was considered to be the end points of the observation. Tumor volume was calculated by the formula: tumor volume = width^2^ × length × π/6 [22]. CO_2_ and cervical dislocation were used for mice euthanasia. Tumors were excised and tumor weight was recorded at the end of the experiments. A part of the tumors from both groups were used for tumor lysate in Western Blot experiments.

### Measurements of blood glucose, insulin and IGF-1

Mice were fasted for 6 hours, and blood glucose concentration was monitored in venous blood drawn from the tail vein using a glucometer (Roche, Basel, Switzerland). At sacrifice, blood samples were collected to measure the serum concentrations of insulin and IGF-1. The serum insulin and IGF-1 were determined by an enzyme immunoassay according to the manufacturer's protocol (R&D Systems, Minneapolis, MN).

### Statistical analysis

SPSS 17.0 software (SPSS Inc., Chicago, IL) was used for statistical analysis. Quantitative results were expressed as the means ± standard deviations (SD). The statistical analysis was performed by Student's t test between two groups or one-way ANOVA for data from multiple groups. P values <0.05 were considered significant.

## Results

### Insulin/IGF-1 promotes colon cancer cells proliferation and cell cycle progression *in vitro*

**Fig 1:**
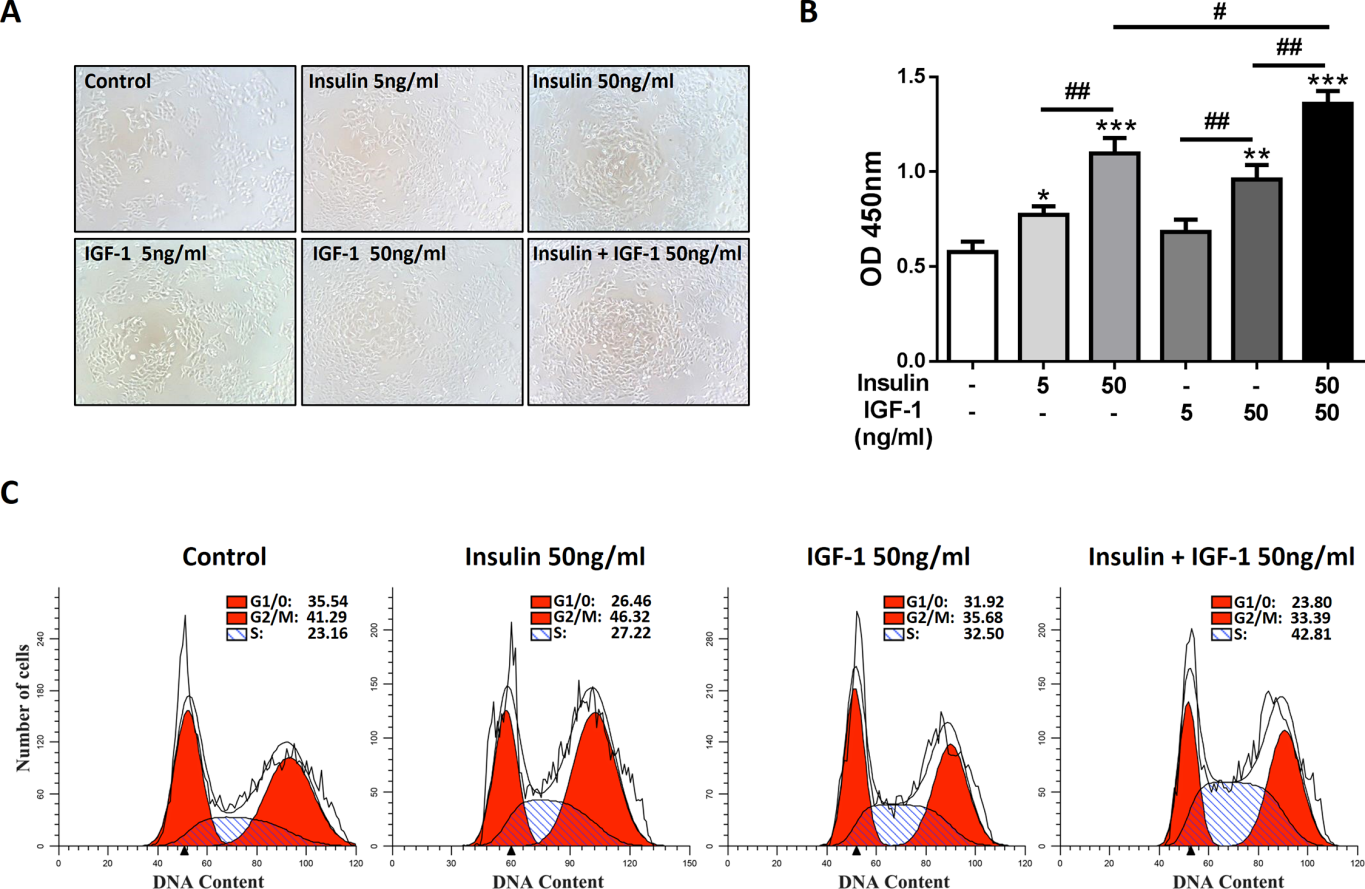
Insulin/IGF-1 promotes colon cancer cells proliferation and cell cycle progression *in vitro*. MC38 cells were cultured with various concentrations of insulin and IGF-1 for 72 hours. Control groups were treated with PBS. (A) Cell morphology was observed. (B) Cells were harvested for proliferation analysis with CCK-8 assay. *P<0.05; **P<0.01; ***P<0.001 versus control, ^#^P<0.05; ^##^P<0.01 between the indicated two groups, n = 3 per group. (C) Cell cycle analysis of insulin/IGF-1 treated cells. DNA content was measured by PI staining on flow cytometry. The percentages of cell cycle phases are shown in each panel. The data shown are representative of three separate experiments.

Previous studies have shown that insulin and IGF-1 have pro-proliferation effects in various cancer cells [13]. We speculated that insulin or IGF-1 got the same effects on MC38 cells, a mouse derived colon cancer cell line. Since blood insulin and IGF-1 levels increased in T2DM patients, combination of insulin and IGF-1 (insulin/IGF-1) was also used to mimic this environment *in vitro*. Indeed, we found that either insulin or IGF-1 promoted the expansion of MC38 cells *in vitro* **(Fig 1A)**. To determine the pro-proliferative effects of insulin and IGF-1, a CCK-8 assay was applied to detect the cell viability. As we expected, either insulin or IGF-1 promoted the proliferation of MC38 cells in a dose dependent manner. Furthermore, the combination of insulin and IGF-1 showed an additive effect in proliferation **(Fig 1B)**.

We next addressed the question whether insulin/IGF-1 promoted cell cycle progression in MC38 cells. For the cell cycle analysis, DNA content was measured by PI staining on flow cytometry after 72 hours of insulin and IGF-1 treatment. Cell cycle analysis showed that insulin/IGF-1 significantly reduced the proportion of MC38 cells in G0/1-phase, and enhanced the proportion of MC38 cells in S-phase **(Fig 1C)**. Thus, these data showed that insulin/IGF-1 promotes colon cancer cell proliferation and cell cycle progression *in vitro*.

### Insulin/IGF-1 inhibits colon cancer cells apoptosis *in vitro*

**Fig 2:**
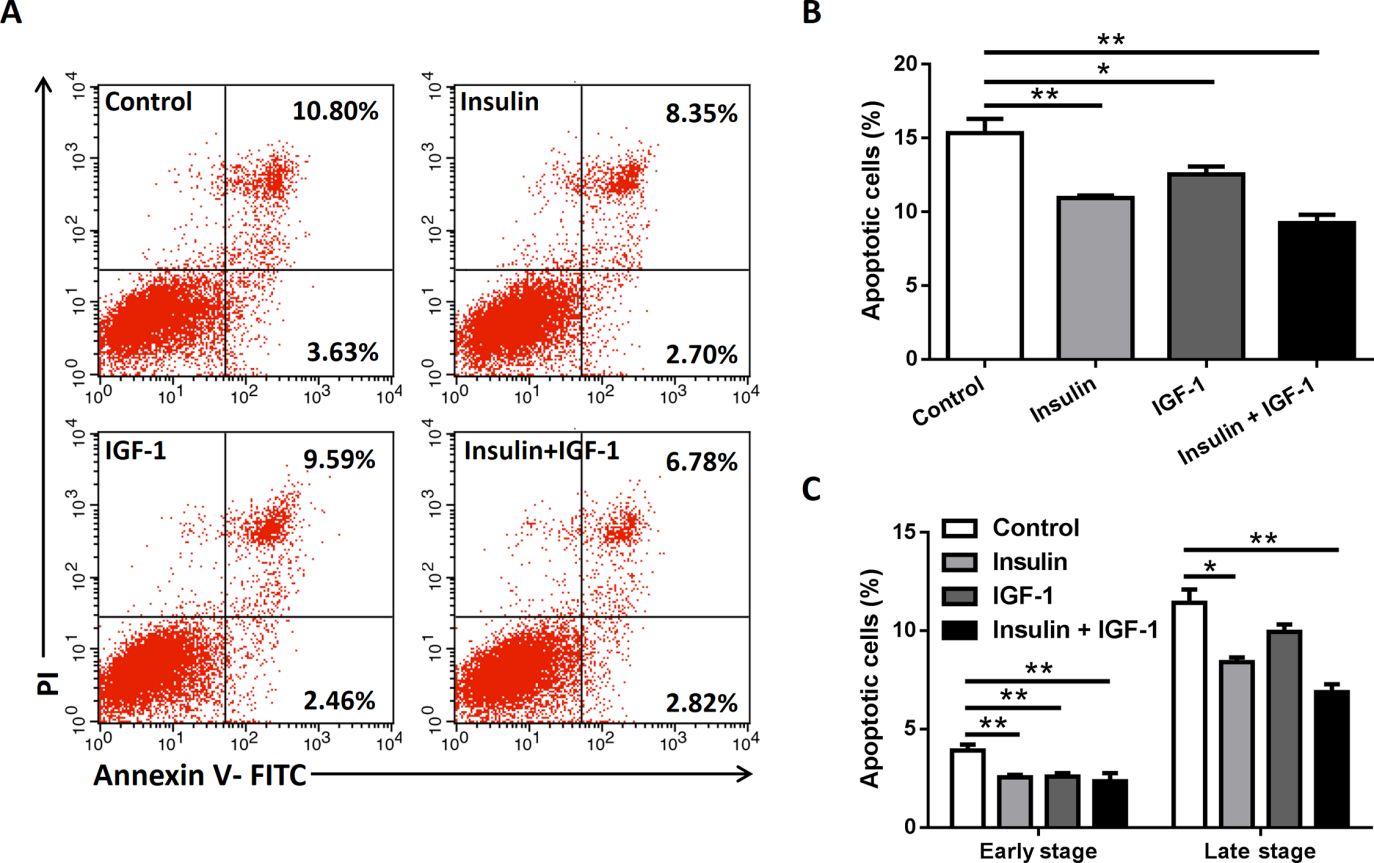
Insulin/IGF-1 inhibits colon cancer cells apoptosis *in vitro*. MC38 cells were cultured with insulin and IGF-1 alone or both together for 72 hours. Control groups were treated with PBS. (A) Cells were harvested for apoptosis analysis by Annexin V and PI staining on flow cytometry. The early stage (Annexin V+/PI-) and late stage (Annexin V+/PI+) apoptotic events were gated. The data shown are representative of three separate experiments. Quantification of total percentage (B) and early/late stage percentage (B) of apoptotic cells after the treatments. *P<0.05; **P<0.01 versus control, n = 3 per group.

Apoptosis plays an important role during cancer growth. To assess whether insulin/IGF-1 affected the apoptosis of MC38 cells *in vitro*, cells were cultured with insulin and IGF-1 alone or both together for 72 hours. Cells were harvested for apoptosis analysis by Annexin V and PI staining on flow cytometry. The apoptosis assay showed that insulin and IGF-1 alone or both together decreased the total apoptotic cells **(Fig 2A and 2B)**. To further identify the stage of apoptosis, the early stage and late stage apoptotic cells were measured too. As expected, all treatments decreased early stage apoptosis and late stage apoptosis, except IGF-1 alone couldn’t affect the late stage apoptosis **(Fig 2A and 2C)**. The results revealed that insulin/IGF-1 inhibits colon cancer cells apoptosis *in vitro*.

### Insulin/IGF-1 activates ERK1/2 and JNK signaling of colon cancer cells *in vitro*

**Fig 3:**
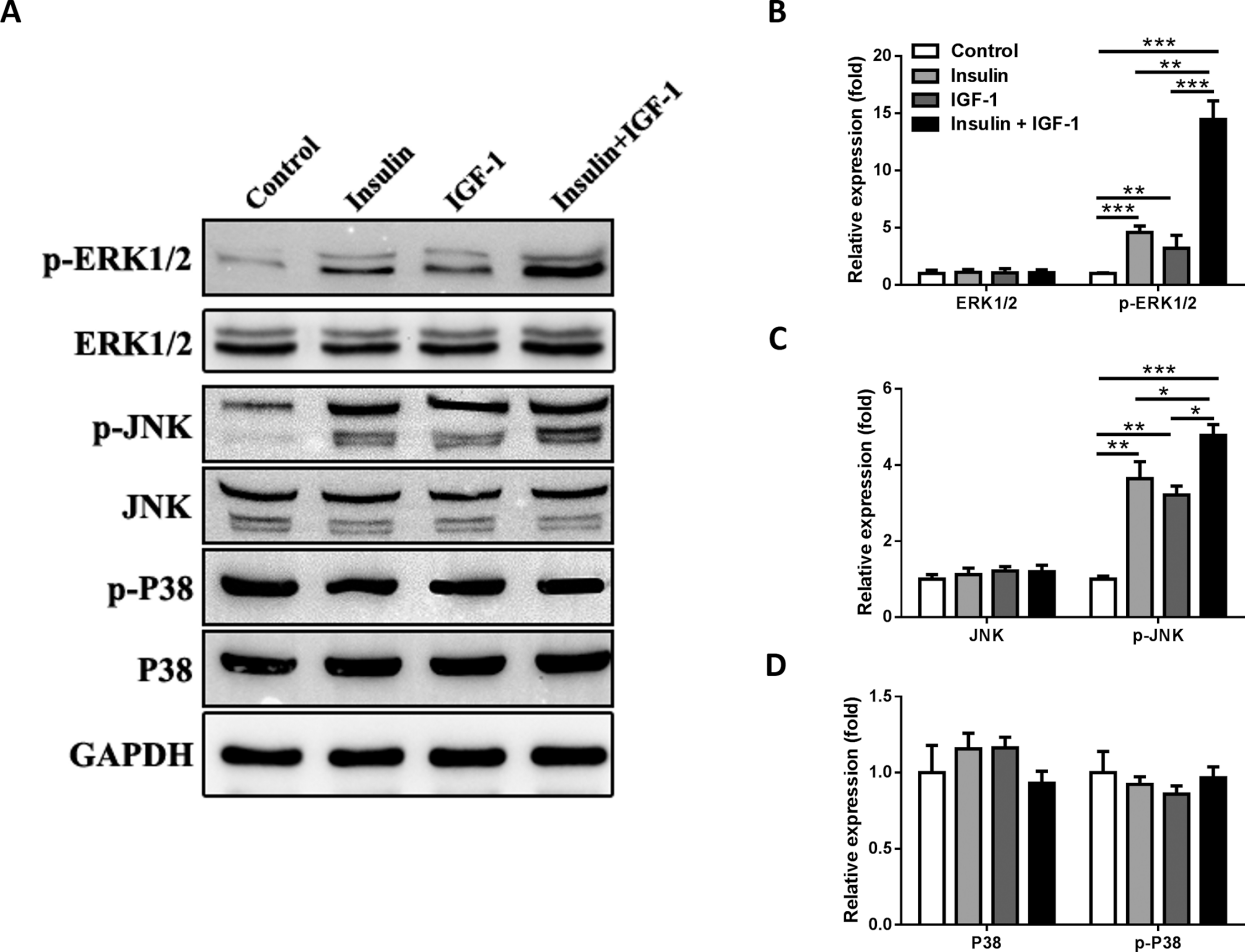
Insulin/IGF-1 activates ERK1/2 and JNK signaling of colon cancer cells *in vitro*. MC38 cells were cultured with insulin and IGF-1 alone or both together for 72 hours and then collected for western blotting analysis. Control groups were treated with PBS. (A) Western blotting analysis of p-ERK1/2, ERK1/2, p-JNK, JNK, p-P38 and P38 protein expression in treated cells. GAPDH served as a loading control. The blots shown are representative of three separate experiments. Semi-quantitation for the expressions of (B) ERK1/2 and pERK1/2, (C) JNK and p-JNK, (D) P38 and p-P38 proteins. Fold changes were normalized by control groups. *P<0.05; **P<0.01; ***P<0.001 versus control, n = 3 per group.

Previous study showed that Ras-Raf-MAPK signal, which acts as downstream of insulin/IGF-1 signaling plays a role in the proliferation of various cancers [18–20]. To determine whether Ras-Raf-MAPK signal involved in insulin/IFG-1sinaling transduction, we detected the 3 main downstream molecules (ERK1/2, JNK and P38) in Ras-Raf-MAPK signal pathway. MC38 cells were cultured for 72 hours with different treatments and harvested for western blot analysis **(Fig 3A)**. We found that insulin, IGF-1 and insulin/IGF-1 increased the phosphorylation of ERK1/2 and JNK, except P38 **(Fig 3B–3D)**. Furthermore, there was additive effect when insulin and IGF-1 were used together **(Fig 3B and 3C)**. Thus, these data showed that insulin/IGF-1 activates ERK1/2 and JNK signaling of colon cancer cells *in vitro*.

### Inhibition of ERK1/2 or JNK signaling abolishes the proliferative and anti-apoptotic effects of insulin/IGF-1 *in vitro*

**Fig 4:**
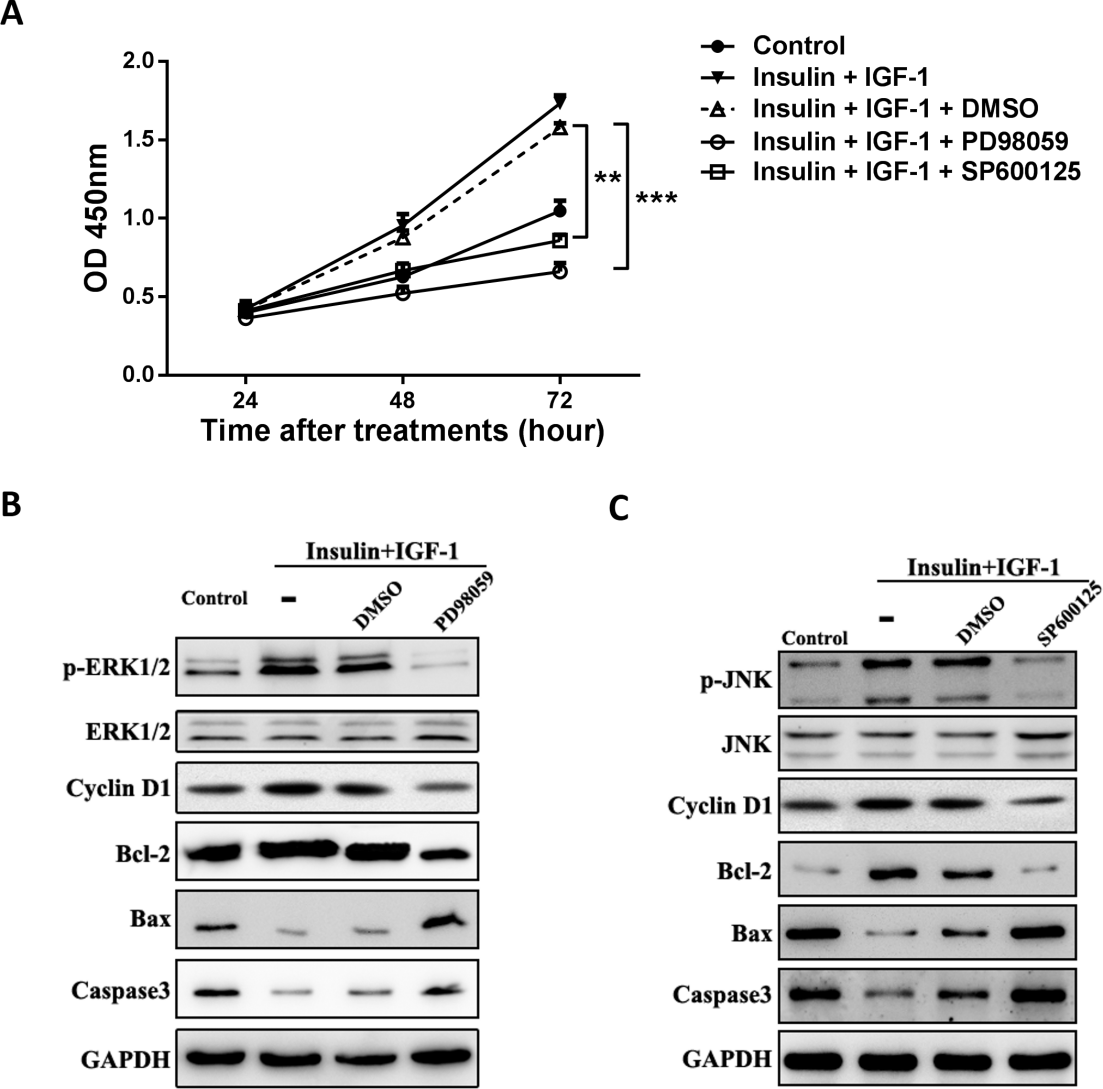
Inhibition of ERK1/2 or JNK signaling abolishes the proliferative and anti-apoptotic effects of insulin/IGF-1 *in vitro*. MC38 cells were cultured with insulin and IGF-1 alone or both together for 72 hours. ERK1/2 inhibitor PD98059 (B), JNK inhibitor SP600125 (C) or their vehicle DMSO added to the cultures when MC38 cells were treated with both insulin and IGF-1. (A) Cells were collected for proliferation analysis with CCK-8 assay at 24, 48 and 72 hour. **P<0.01; ***P<0.001 between the indicated two groups, n = 3 per group. (B and C) Western blotting analysis for p-ERK1/2, ERK1/2, p-JNK, JNK, Cyclin D1, Bcl2, Bax and Caspase3 protein expression in the treated MC38 cells. GAPDH served as a loading control. The blots shown are representative of three separate experiments.

We next assessed whether the effects of insulin/IGF-1 signal was ERK1/2 and JNK dependent. MC38 cells were treated with insulin/IGF-1 with or without 40 μM ERK1/2 inhibitor PD98059 or 20 μM JNK inhibitor SP600125 for 24, 48 or 72 hours. The proliferation rates were measured by using CCK-8 kit. As shown in **Fig 4A**, the proliferative effect of insulin/IGF-1 was abolished by either PD98059 or SP600125. We also detected the effects of ERK1/2 inhibitor **(Fig 4B)** and JNK inhibitor **(Fig 4C)** in phosphorylation, apoptosis and proliferation with western blot. The combined insulin/IGF-1 dramatically increased the expression of anti-apoptotic protein Bcl-2, proliferative protein Cyclin D1 and decreased the expression of apoptotic protein Bax and Caspase 3. Treated with either PD98059 or SP600125 could reverse these effects. The results showed that inhibition of ERK1/2 or JNK signaling abolishes the proliferative and anti-apoptotic effects of insulin/IGF-1 *in vitro*.

### Endogenous insulin and IGF-1 are increased in mouse type 2 diabetes model

**Fig 5:**
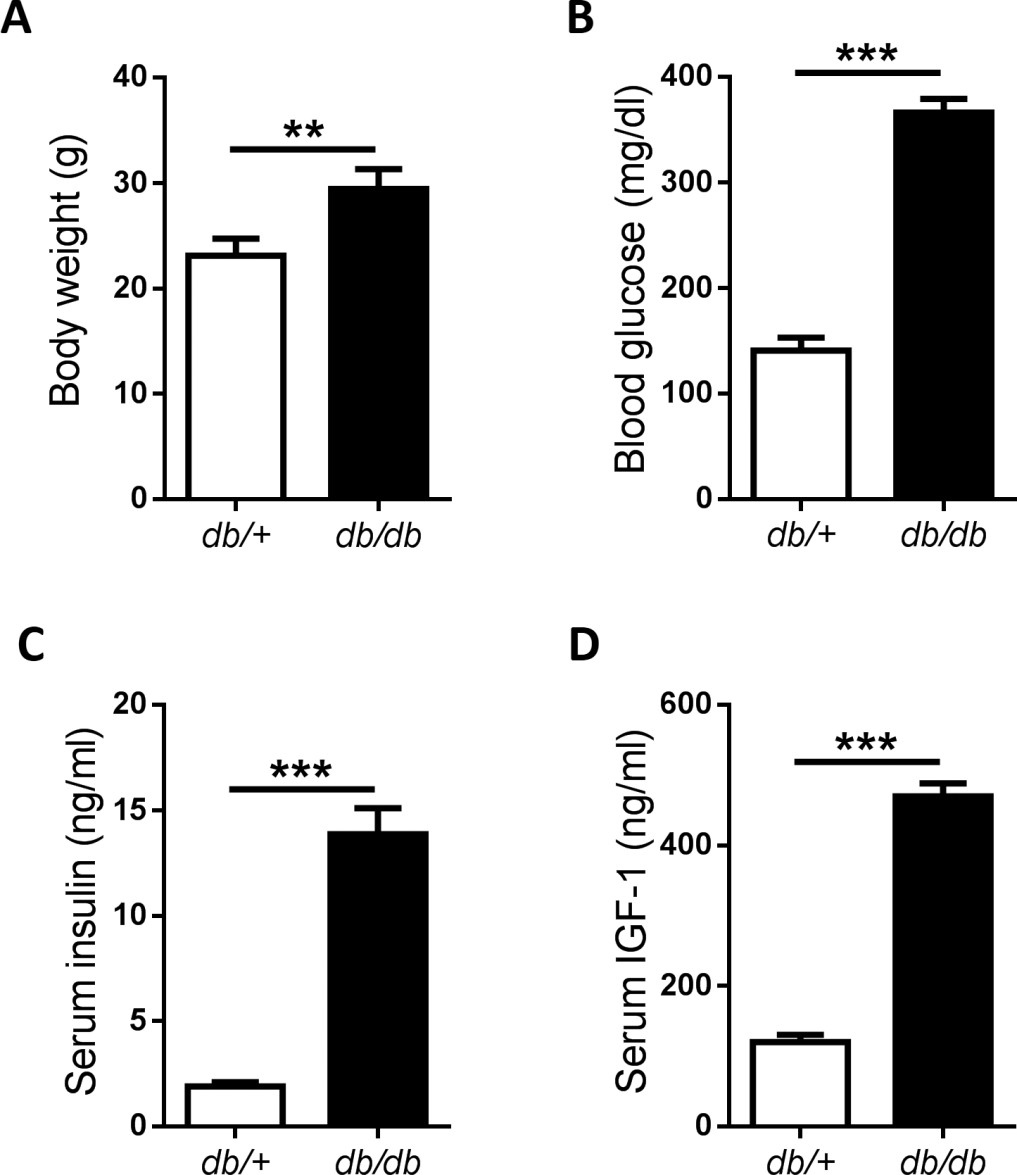
Establishment of type 2 diabetes model with *db/db* mice. Male *db/db* mice were used as mouse type 2 diabetes models, while *db/+* littermates as normal controls. Body weight (A), blood glucose (B), insulin (C) and IGF-1 (D) were determined before MC38 cells injection at 8^th^ week. *P<0.05; ***P<0.001, n = 5 per group.

Eight-week-old male *db/db* mice were used to establish a spontaneous type 2 diabetes model. While their *db/+* littermates were used as normal controls. At the beginning of the experiments, the body weight and blood glucose were tested to confirme whether the diabetic model were successful. We found that the *db/db* mice were obese compared to their *db/+* littermates which got the normal weight **(Fig 5A)**. The fasting blood glucose in *db/db* mice was dramatically elevated **(Fig 5B)**. We then determined whether the serum insulin and IGF-1 were changed in these mice. Serum insulin was increased in *db/db* mice as expected **(Fig 5C)**. What’s more, the serum IGF-1 in *db/db* mice was also increased significantly **(Fig 5D)**. These results indicated that *db/db* mice are ideal models for type 2 diabetes with high level of insulin and IGF-1.

### Endogenous insulin/IGF-1 accelerates colon tumor growth in mouse type 2 diabetes model

**Fig 6:**
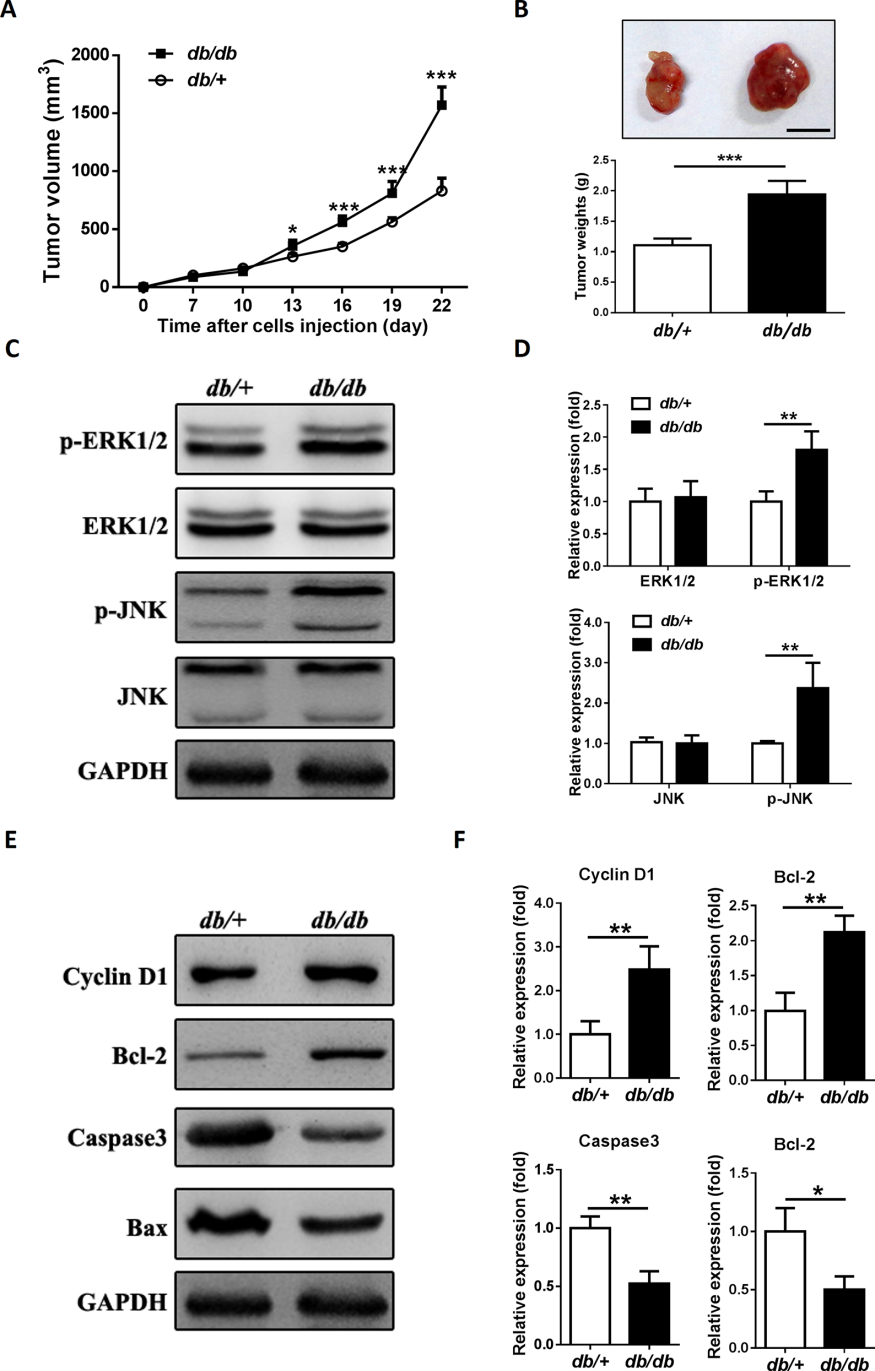
Endogenous insulin/IGF-1 accelerates colon tumor growth in a mouse type 2 diabetes model. 2 × 10^6^ MC38 cells suspended in 0.1 ml of PBS were subcutaneously injected into the *db/db* and *db/+* mice to initiate tumor growth *in vivo*. (A) Tumor size was measured every 3 days. *P<0.05; ***P<0.001. (B) The tumors were excised and weighted 3 weeks after cell injection. ***P< 0.001. A representative tumor mass from each group was shown in the inset (scale bar = 1cm). (C and E) Western blotting analysis of p-ERK1/2, ERK1/2, p-JNK, JNK, Cyclin D1, Bcl-2, Bax and Caspase3 protein expression in tumors. GAPDH served as a loading control. The blots shown are representative of three separate experiments. (D and F) Semi-quantitation for the expressions of ERK1/2 and pERK1/2, JNK and p-JNK, Cyclin D1, Bcl-2, Bax and Caspase3 protein. Fold changes were normalized by control groups. *P<0.05; **P<0.01 versus control, n = 3 per group.

To further validate the effects of endogenous insulin/IGF-1 in the growth of colon tumor, MC38 cells were subcutaneously injected into the *db/db* or *db/+* mice to initiate tumor growth. Tumor volumes were measured at different time points after inoculation, and tumor weights were measured after sacrifice. We found that tumor growth was faster, and tumor weight was heavier in *db/db* mice than *db/+* mice **(Fig 6A and 6B)**. Next we detected the phosphorylation of ERK1/2 and JNK, Cyclin D1, Bcl-2, Caspase 3 and Bax in tumor tissue. Both p-ERK1/2 and p-JNK expressions were higher in *db/db* mice **(Fig 6C and 6D)**. The expressions of Cyclin D1 and Bcl-2 were increased, while the expressions of Bax and Caspase 3 were decreased in *db/db* mice, which was consistent with the observations *in vitro* **(Fig 6E and 6F)**. The results showed that endogenous insulin/IGF-1 accelerates colon tumor growth in a mouse type 2 diabetes model.

### Inhibition of ERK1/2 or JNK signaling suppresses the development of colon tumor in mouse type 2 diabetes model

**Fig 7:**
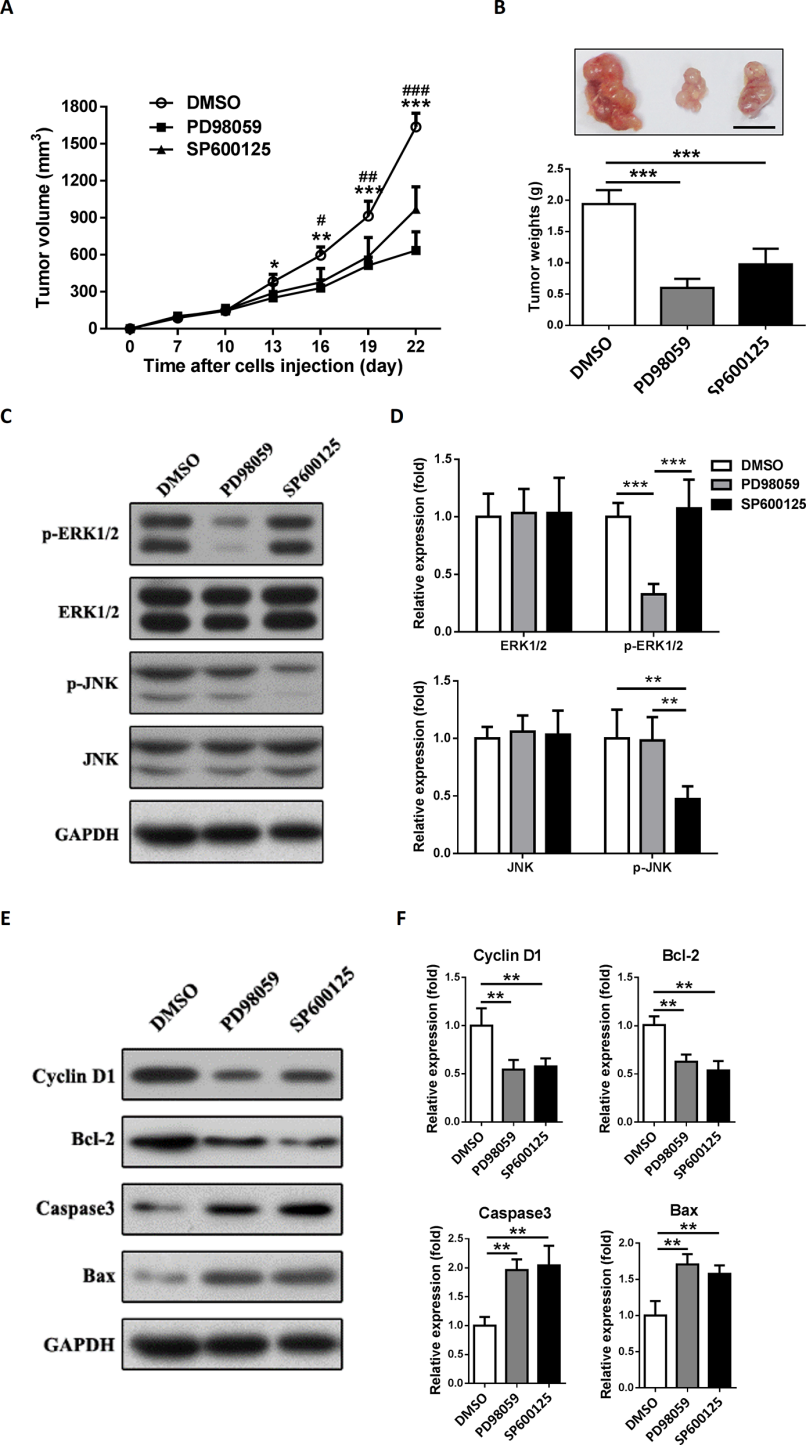
Inhibition of ERK1/2 or JNK signaling suppresses the development of colon tumor in type 2 diabetes model. 2 × 10^6^ MC38 cells suspended in 0.1 ml of PBS were subcutaneously injected into the *db/db* mice to initiate tumor growth *in vivo*. 10 mg/kg PD98059 or 30 mg/kg SP600125 was administered intraperitoneally every 3 days when tumor volume reached 100mm^3^. 1% DMSO was used as control treatment. (A) Tumor size was measured every 3 days. *P<0.05; ***P<0.001. (B) The tumors were excised and weighted 3 weeks after cell injection. ***P<0.001. A representative tumor mass from each group was shown in the inset (scale bar = 1cm). (C and E) Western blotting analysis of p-ERK1/2, ERK1/2, p-JNK, JNK, Cyclin D1, Bcl-2, Bax and Caspase3 protein expression in tumors. GAPDH served as a loading control. The blots shown are representative of three separate experiments. (D and F) Semi-quantitation for the expressions of ERK1/2 and pERK1/2, JNK and p-JNK, Cyclin D1, Bcl-2, Bax and Caspase3 protein. Fold changes were normalized by control groups. **P<0.01; ***P<0.001 versus control, n = 3 per group.

At the end of this study, to figure out whether the development of colon tumor in *db/db* mice was ERK1/2 or JNK dependent, we used ERK1/2 or JNK inhibitor to treat the colon tumor bearing *db/db* mice. We found that the effect of ERK1/2 or JNK inhibitor was dose-dependent and 10 mg/kg PD98059 or 30 mg/kg SP600125 showed a better effect for blocking tumorous ERK1/2 or JNK signaling respectively *in vivo* **(S1 Fig)**. As we expected, PD98059 or SP600125 showed a significant suppression in colon tumor growth *in vivo*, which was consistent with the results found *in vitro* **(Fig 7A)**. The tumor mass measurements at the end of experiments showed the similar results **(Fig 7B)**.

We also detected the expressions of targeted and downstream proteins after PD98059 or SP600125 treatment in this model **(Fig 7C–7F)**. As we expected, PD98059 and SP600125 specifically blocked ERK1/2 and JNK respectively *in vivo* **(Fig 7C and 7D)**. Both PD98059 and SP600125 reduced the expression of Cyclin D1 and Bcl-2 but increased the expression of Caspase 3 and Bax **(Fig 7E and 7F)**. These results confirmed that inhibition of ERK1/2 or JNK signaling suppresses the development of colon tumor in mouse type 2 diabetes model.

## Discussion

In the present study, we observed that insulin and IGF-1, act as mitogens of colon cancer cells, activate the ERK1/2 and JNK MAPK signaling, resulting in cell cycle acceleration, cell growth and anti-apoptosis in colon cancer MC38 cells. Moreover, we confirmed these findings *in vivo* by establishing a colon tumor allograft model with T2DM.

Diabetes is characterized by defects in glucose homeostasis and insulin function. T2DM, which is characterized by insulin resistance and high insulin levels, accounts for 90–95% diabetes mellitus [23]. Decades of epidemiological evidence showed that T2DM was associated with an increased risk of cancer at different sites, including colorectum, liver, pancreas, breast and bladder [24]. Our previous study found that T2DM might be one of the important pathogenic risk factors for colorectal cancer in Chinese [25]. However, there were also some investigators claimed that these associations could be caused by biases in the literature [26, 27]. A recent umbrella review which assessed associations between T2DM and risk of developing cancer in 20 sites, showed that only colorectal, breast, intrahepatic cholangiocarcinoma, and endometrial cancer had robust evidence to substantiate the risk of developing cancer without hints of bias [28].

The mechanisms of T2DM promotes the development of cancer are yet to be investigated, including hyperinsulinemia, high level of IGF-1 and hyperglycemia in T2DM [29]. Our previous studies showed that insulin glargine promoted the growth of human colon cancer (HCT-116 and SW480 cells) and breast cancer (MCF-7 cells) *in vitro* [30, 31]. What’s more, glargine insulin treatment was believed to increase the risk of developing colon cancer [32]. Additionally, hyperinsulinemia also leads to increase the level of bioactive IGF-1, which is a potent mitogen that can result in carcinogenesis [33]. However, a randomized-controlled trial did not support the hypothesis that hyperglycemia was related to increased cancer risk [34]. Hence, hyperinsulinemia and high level of IGF-1 may be its main mechanisms. The results of our study support this hypothesis. We found that the combination of insulin and IGF-1 significantly promoted the growth and the cell cycle of murine colon cancer MC38 cells than single use of insulin or IGF-1 *in vitro*. This result also showed that the insulin/IGF-1 had an additive effect in MC38 cells proliferation.

MAPK signaling pathways, which convert extracellular signals into specific cellular responses through series of phosphorylation events, play an important role in colon cancer development and metastasis [35]. ERK1/2, P38 and JNK are the 3 major MAPK families found activated in colorectal cancer. Previous studies suggested that the ERK1/2 pathway, but not the JNK or the P38 pathway, is a major regulator of cell proliferation in colorectal cancer [36]. The other two MAPK pathways, the P38 and JNK, mediate apoptosis and inflammatory response [36]. However, the effects of JNK activation may be diverse in regulation of apoptosis when the cell receiving different stimulation. For instant, growth factors lead to proliferation and anti-apoptosis via JNK pathway, whereas the inflammatory cytokines (e.g. TNF-α) lead to apoptosis [37]. It has been widely accepted that insulin or IGF-1 promotes the proliferation of various cancers by activating MAPK signalings [18–20]. Our present study clearly showed that insulin/IGF-1 activated ERK1/2 and JNK MAPK but not P38 MAPK signaling in proliferation of MC38 cells. The inhibition of ERK1/2 or JNK showed that both of these MAPK signalings contributed to proliferation and anti-apoptosis. However, Carlson et al. reported that insulin didn’t enhance ERK1/2, JNK or P38 phosphorylation in adipocytes from T2MD patients [38]. While Gogg et al. showed that MAPK signaling (ERK1/2) was increased but the insulin signaling was impaired in subcutaneous microvascular endothelial cells from T2MD patients [39]. The studies mentioned above and our results indicated that the activation and effects of MAPK signaling by insulin or IGF-1 in different cell lines may be various.

Next, we intended to confirm our hypothesis with a mouse T2DM model *in vivo*. The lack of reliable animal models which mimic human T2DM has delayed the investigations of specific mechanisms involved in the interactions between T2DM and cancer development. During these decades, several mouse T2DM models were introduced into diabetic studies. There are 2 types of mouse T2DM model: induced and spontaneous T2DM models [40]. High fat diet plus low dose of streptozotocin is a frequently-used approach to induced T2DM in mice. However, long time required for induction, unstable hyperinsulinemia and large variation are the limitations of this model. [41]. The leptin receptor knockout mice (*db/db* mice) are relatively suitable for spontaneous T2DM model [42]. In the current study, we found that the blood glucose, serum insulin and IGF-1 were extremely higher in *db/db* mice than *db/+* mice at 8-week old. These parameters were correlated with T2DM patients. Besides, both *db/db* mice and colon cancer MC38 cells we used were with C57BL/6 background, which made it possible for us to establish a MC38 colon tumor allograft in *db/db* mice. Hence, *db/db* mice became the ideal spontaneous T2DM model in our study. Kazuya et al. reported that *db/db* mice were susceptible to develop azoxymethane-induced dysplastic and early neoplastic lesions in colon [43]. So far as we know, our study is the first time to establish colon tumor allograft in a spontaneous T2DM model with *db/db* mice. Here, we showed that the growth of MC38 allografts in *db/db* mice was accelerated. Excessive activation the ERK1/2 and JNK MAPK signaling of tumor allograft was found in the *db/db* mice, which contributed to the proliferation and anti-apoptosis in MC38 cells. Unlike the *in vitro* study, the serum level of IGF-1 is approximately more than 30 times higher compared to Insulin in *db/db* mice **(Fig 6C and 6D)**. This may be a limitation for mimicking the *in vitro* studies with current model. Previous studies have already showed that ERK1/2 or JNK inhibitor suppressed colon cancer development [44]. In the current study, we also demonstrated that inhibition of ERK1/2 or JNK could abolish the progression of colon tumor allograft in *db/db* mice.

In conclusion, our study suggests that the activation of ERK1/2 and JNK MAPK signaling by insulin and IGF-1, at least in part, is responsible for the development of colon cancer with T2DM. New insulin independent anti-diabetic therapy might be benefit to colon cancer with T2DM.

## Supporting Information

S1 Fig The effects of PD98059 and SP600125 in the MC38 cells-bearing model with *db/db* mice. (DOCX)
