# Supplementary material for: The Activation of ERK1/2 and JNK MAPK Signaling by Insulin/IGF-1 Is Responsible for the Development of Colon Cancer with Type 2 Diabetes Mellitus
Source: PLoS One. 2016 Feb 22;11(2):e0149822. doi: 10.1371/journal.pone.0149822 (PMC4763097; doi:10.1371/journal.pone.0149822)
Supplement: S1 Fig — (DOCX) [file pone.0149822.s001.docx]

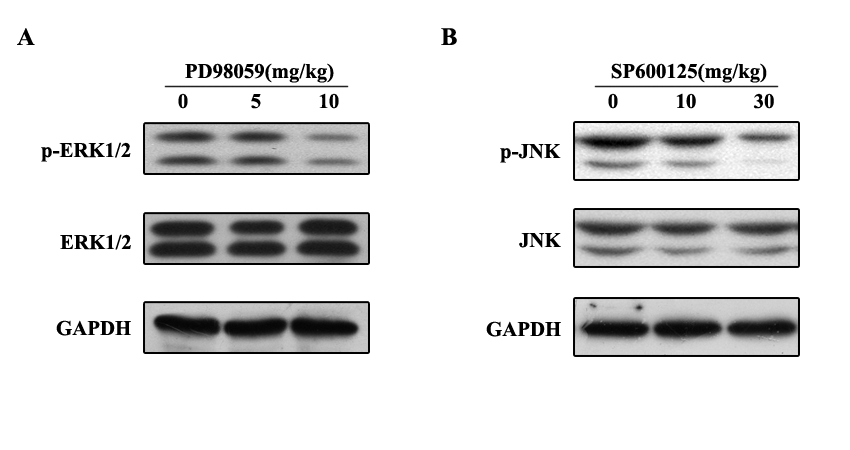


**S1 Fig. The effects of PD98059 and SP600125 in the MC38 cells-bearing model with *db/db* mice.** 2 × 10^6^ MC38 cells suspended in 0.1 ml of PBS were subcutaneously injected into the *db/db* mice to initiate tumor growth *in vivo*. Different concentrations of PD98059 (0, 5 or 10mg/kg) or SP600125 (0, 10 or 30mg/kg) was administered intraperitoneally every 3 days when tumor volume reached 100mm^3^. Tumors were harvested after 1 week later, and the expressions of p-ERK1/2 and ERK1/2 in PD98059 treated group (A), p-JNK and JNK in SP600125 treated group (B) were detested by western blotting analysis. GAPDH served as a loading control. The blots shown are representative of three separate experiments.
